# Successful treatment of intractable pseudomeningocele with FXIII deficiency by surgery and FXIII replacement therapy: A case report

**DOI:** 10.1016/j.ijscr.2022.106851

**Published:** 2022-02-26

**Authors:** Nobutoshi Takamatsu, Hiroaki Manabe, Kazuma Wada, Tetsuya Hirano, Takashi Chikawa, Koichi Sairyo

**Affiliations:** a Department of Orthopedics, Institute of Biomedical Science, Tokushima University Graduate School, 3-18-15 Kuramoto, Tokushima City, Tokushima 770-8503, Japan; b Department of Orthopedics, Tokushima Prefecture Naruto Hospital, 32 Muya, Naruto city, Tokushima 772-8503, Japan

**Keywords:** Cerebrospinal fluid leakage, Pseudomeningocele, Surgery FXIII deficiency, FXIII replacement, Case report

## Abstract

Pseudomeningocele is an extradural cystic collection of cerebrospinal fluid (CSF) and is rare and typically asymptomatic. However, pseudomeningocele is sometimes associated with symptoms. Whether symptomatic pseudomeningocele is best treated conservatively or surgically remains controversial. Factor XIII (FXIII) is a blood coagulation factor that also promotes fibroblast proliferation during wound healing. Although treatment of postsurgical CSF leakage with FXIII has been reported, there have been no reports on surgical treatment and FXIII replacement therapy of pseudomeningocele with FXIII deficiency. We report a case of pseudomeningocele with FXIII deficiency that was successfully treated by surgery and FXIII replacement therapy. The patient presented with symptoms of intracranial hypotension syndrome that had started a few months after laminectomy for thoracic ossification of the ligamentum flavum 2 years earlier. Magnetic resonance imaging and delayed computed tomography myelography confirmed a diagnosis of pseudomeningocele. Epidural blood patch treatment was performed twice but did not result in improvement. Furthermore, the FXIII level decreased to 56%, so the patient was also diagnosed as having acquired FXIII deficiency. We elected to treat the patient by surgery with FXIII replacement therapy. The dural injury was repaired using an artificial dura mater patch, fibrin glue, and polyglycolic acid sheets. The FXIII level was 74%–135% during the perioperative period. The patient had a good postoperative course. Postoperative magnetic resonance images showed resolution of the pseudomeningocele. There was no recurrence during 6 months of follow-up. Perioperative FXIII replacement may be a useful treatment for pseudomeningocele with FXIII deficiency.

## Highlights

• Pseudomeningocele is an extradural cystic collection of cerebrospinal fluid.
• Whether symptomatic pseudomeningocele is best treated remains controversial.
• Factor XIII is a blood coagulation factor that also promotes fibroblast proliferation.
• There have been no reports on surgical treatment and FXIII replacement therapy of pseudomeningocele with FXIII deficiency.
• We report a case of pseudomeningocele treated by surgery and FXIII replacement therapy.

## 1 Introduction

Pseudomeningocele is an extradural cystic collection of cerebrospinal fluid (CSF) that communicates with the arachnoid space via a dural tear [1], [2]. Most pseudomeningoceles are asymptomatic and resolve spontaneously without treatment. However, some produce symptoms, and the decision as to whether they should be treated conservatively or surgically is sometimes difficult and remains controversial.

Factor XIII (FXIII) is a blood coagulation factor that not only catalyzes the formation of cross-links between fibrin molecules during coagulation but also promotes fibroblast proliferation during wound healing [3], [4], [5]. Although treatment of postsurgical cerebrospinal fluid (CSF) leakage by intravenous FXIII has been reported [6], [7], there are no reports on treatment of pseudomeningocele with FXIII deficiency by surgery and FXIII replacement therapy. Pseudomeningocele and CSF leakage are caused by similar mechanisms and can be considered as a continuum. Therefore, FXIII may also be an effective treatment for pseudomeningocele [1]. Herein, we describe a case of intractable pseudomeningocele with FXIII deficiency that was treated successfully by surgery and FXIII replacement therapy. This case report has been reported in line with the SCARE Criteria [8].

## 2 Case report

**Fig. 1:**
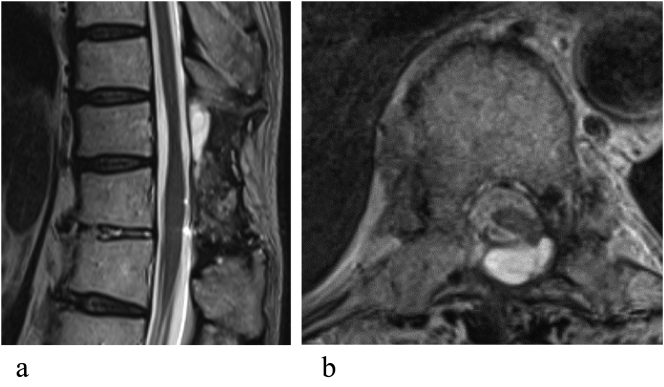
T2-weighted magnetic resonance scans show an extradural cystic lesion associated with laminectomy at T10–11. (a) Sagittal view. (b) Axial view.

**Fig. 2:**
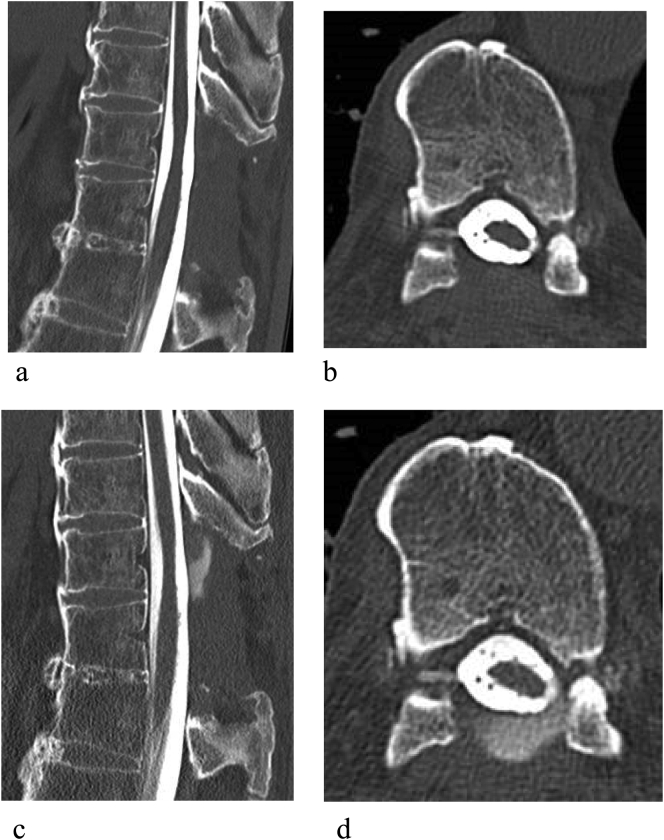
Computed tomography myelograms at T10–11. (a) Sagittal view. (b) Axial view. Delayed computed tomography myelograms show flow of contrast into the lesion at T10–11. (c) Sagittal view. (d) Axial view.

A 80-year old man had a history of difficult healing of a foot wound but no background of excessive bleeding. Past medical history was reflux esophagitis and the medication included vonoprazan fumarate. The patient had no allergies and the family did not have history of excessive bleeding or a known coagulation disorder. The patient had undergone laminectomy at T3–5 and T10–11 for thoracic ossification of the ligamentum flavum at another hospital 2 years earlier. CSF leakage occurred intraoperatively as a result of a 10-mm × 4-mm dural defect at T10–11 with ossification. The defect was sutured and repaired using fibrin glue and polyglycolic acid sheets. The left leg pain and numbness improved immediately after surgery. However, within a few months after surgery, the patient started to complain of several symptoms, including positional headache, nausea, photophobia, and tinnitus. The patient consulted an otolaryngologist and a neurosurgeon considering cochlear or cerebellar disorders but no abnormalities were detected. The symptoms gradually worsened and the patient was referred to our department for further examination and treatment. Magnetic resonance imaging (MRI) revealed an extradural cystic lesion above the laminectomy at T10–11. The lesion showed low signal intensity on T1-weighted images and high signal intensity on T2-weighted images (Fig. 1). Persistent CSF leakage was suspected. Delayed computed tomography (CT) myelography revealed flow of contrast into the lesion. The diagnosis was pseudomeningocele (Fig. 2A, B). The symptoms continued despite two attempts at epidural blood patching and the pseudomeningocele was still present on MRI scans. In view of the intractable pseudomeningocele, we measured the FXIII level and found it to be decreased to 56% (reference range, 70%–130%) although the inhibitor status was negative. Therefore, the patient was also diagnosed to have acquired FXIII deficiency. Surgery was performed with administration of FXIII replacement therapy.

## 3 Surgical intervention with FXIII replacement therapy

After administration of 1200-U of fibrogammin P, the FXIII level increased to 107%. The orthopedic spine surgeon in our hospital then performed additional partial laminectomy at T9 and found the same dural injury that caused the leak of CSF at T10. Again, the surgeon sutured the dural injury with 6-0 polypropylene and sealed it using an artificial dura mater patch. Finally, the surgeon applied fibrin glue and polyglycolic acid sheets. The surgeon confirmed that there was no CSF leakage at this or any other site under positive pressure ventilation. The total operating time was 138 min and the estimated blood loss was 50 ml. No complications, such as dural tear, hematoma, or infection, were encountered during or after the surgery. Further 1200-U of fibrogammin P were administered daily on the first four postoperative days while monitoring the FXIII level, which remained at 74%–135% during the perioperative period.

## 4 Postoperative course

**Fig. 3:**
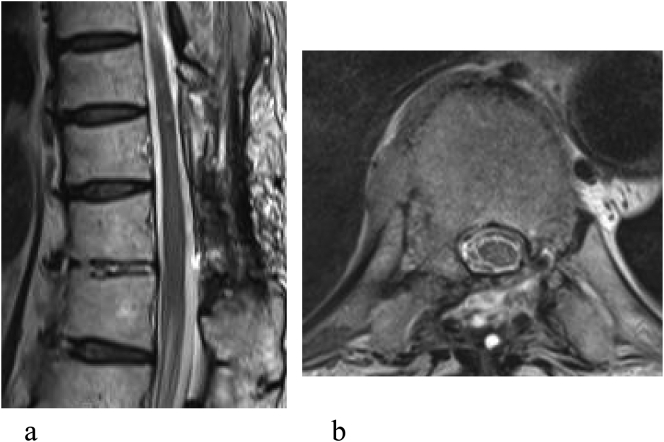
Postoperative T2-weighted magnetic resonance scan showing resolution of the pseudomeningocele. (a) Sagittal view. (b) Axial view.

The patient had a favorable postoperative course with improvement of the symptoms. Wound healing was more rapid than after the previous surgery. The patient was discharged 12 days after surgery and returned to the usual activities of daily living. Postoperative MRI showed resolution of the pseudomeningocele (Fig. 3). There has been no recurrence during 6 months of follow-up.

## 5 Discussion

### 5.1 Pseudomeningocele

Pseudomeningocele and CSF leakage after surgery are rare and usually iatrogenic. Swanson et al. reported that pseudomeningocele occurred after laminectomy in 0.068% of cases and Teplick et al. reported an incidence of 2% [9], [10]. Pseudomeningocele is diagnosed mainly by MRI. Delayed CT myelography in particular helps to visualize the location of the pseudomeningocele in relation to the surgical defect and to differentiate a congenital defect, such as arachnoid cyst, arachnoid diverticulum, or meningeal cyst, and tumors, such as neurinoma and cavernous angioma [11].

Although most pseudomeningoceles are asymptomatic, some may be associated with symptoms, including intracranial hypotension syndrome, radiculopathy, and myelopathy [1]. Our present case had intracranial hypotension syndrome without myelopathy. Mild symptomatic pseudomeningocele may be treated conservatively by bed rest, drainage of spinal CSF, and blood patches [12], [13], [14], [15]. Although spinal CSF drainage might be effective within a few weeks of spinal surgery, our patient was several months out from surgery [12]. Delayed CT myelography suggested a fistula site, so we recommended treatment with an epidural blood patch. However, the pseudomeningocele did not improve after two epidural blood patches.

Surgery is generally recommended for patients in whom pseudomeningocele produces severe symptoms and in those in whom conservative treatment is ineffective [12]. A fascia patch, watertight sutures, and fibrin glue may be helpful repair methods [2], [11], [12], [16], [17], [18]. To achieve watertight closure of the dural injury in our case, we also used a dura mater patch, fibrin glue, and polyglycolic acid sheets after suturing the injury. While appropriate treatment of pseudomeningocele continues to be controversial, our patient had a good postoperative course.

### 5.2 Relationship between pseudomeningocele and FXIII

FXIII is the final enzyme in the coagulation cascade and has unique chemical properties and physiological functions, including catalyzing the intermolecular cross-linking of fibrin polymers, which increases the stability of the fibrin clot [3], [19]. However, FXIII has been identified to have additional extracellular and intracellular roles in wound healing and tissue repair [4]. There have been reports of intravenous FXIII being used successfully to treat postsurgical CSF leakage [6], [7], which suggests that FXIII deficiency may be one of the causes of pseudomeningocele.

### 5.3 Diagnosis of FXIII deficiency

Congenital FXIII deficiency leads to a lifelong bleeding tendency and should be suspected when the patient is a child. However, acquired FXIII deficiency is difficult to diagnose, as in the present case. The usual cause is hyperconsumption or hyposynthesis, and affected patients rarely have bleeding problems in everyday life. Moreover, routine screening coagulation studies will be normal, including the platelet count, prothrombin time, partial thromboplastin time, platelet function assays, fibrinogen, and thrombin clotting time. We suspected FXIII deficiency in our patient because the pseudomeningocele was intractable. FXIII deficiency should be considered in a patient with pseudomeningocele after surgery.

### 5.4 FXIII replacement therapy

FXIII replacement therapy was approved in Japan for suture insufficiency and fistula associated with decreased blood coagulation factor in 1985 and for acquired FXIII deficiency in 2013. The recommendation is to keep FXIII at 50%–100% or more in patients with FXIII deficiency during the perioperative period depending on the degree of invasiveness of surgery [5]. Although the FXIII level that is sufficient in terms of safety is unclear, we maintained it at 74%–135% in our patient during the perioperative period and encountered no complications. However, immunosuppressive therapy may have been needed in addition to FXIII replacement therapy if he had been found to have the inhibitor.

## 6 Conclusion

To our knowledge, this is the first report of successful treatment of intractable pseudomeningocele by surgery and FXIII replacement in a patient with FXIII deficiency. The FXIII level should be measured in a patient with pseudomeningocele after surgery. Perioperative FXIII replacement therapy is recommended for patients with FXIII deficiency.

## Informed consent

Written informed consent was obtained from the patient for publication of this case report and accompanying images.

## Sources of funding

All authors have no sources of funding.

## Ethical approval

This is a case report with an analysis of the literature and then no Ethical Approval was necessary.

## Research registration

Not applicable.

## Guarantor

Dr. Nobutoshi Takamatsu.

## Statement of provenance and peer-review statement

Provenance and peer review Not commissioned, externally peer-reviewed.

## CRediT authorship contribution statement

Dr. Hiroaki Manabe, Dr. Kazuma Wada, Dr. Tetsuya Hirano, Dr. Takashi Chikawa and Dr. Koichi Sairyo were involved in the management of this patient.

## Declaration of competing interest

All authors declare no conflicts of interest in relation to manuscript.

## References

[1] Couture D., Branch C.L. (2003). Spinal pseudomeningocele and cerebrospinal fluid fistulas. Neurosurg. Focus..

[2] Solomon P., Sekharappa V., Krishnan V., David K.S. (2013). Spontaneous resolution of postoperative lumbar pseudomeningoceles: a report of four cases. Indian J Orthop.

[3] Sakata Y., Aoki N. (1980). Cross-linking of alpha 2-plasmin inhibitor to fibrinstabilizing factor. J. Clin. Invest..

[4] RichardsonVR Cordell P., Standeven K.F., Carter A.M. (2013). Substrates of factor XIII-A: roles in thrombosis and wound healing. Clin. Sci..

[5] Bolton-Maggs P.H.B., Perry D.J., Chalmers E.A. (2004 Sep). The rare coagulation disorders – review with guidelines for management from the United Kingdom haemophilia Centre Doctors' organisation. Haemophilia.

[6] Kawamura A., Tamaki N., Yonezawa K., Nakamura M., Asada M. (1997). Effect of factor XIII on intractable CSF leakage after a transpetrosal-approach operation: a case report. No Shinkei Geka.

[7] Nakano M., Araki Y., Kanamori F. (2020). Thrombosis of great vein of Galen caused by factor XIII concentrate: a case report. Jpn. J. Stroke.

[8] Agha R.A., Franchi T., Sohrabi C., Mathew G. (2020). For the SCARE group: the SCARE 2020 guideline: updating consensus surgical CAse REport (SCARE) guidelines. Int. J. Surg..

[9] Swanson H.S., Fincher E.F. (1947). Extradural arachnoidal cysts of traumatic origin. J. Neurosurg..

[10] Teplick J.G., Peyster R.G., Teplick S.K., Goodman L.R., Haskin M.E. (1983). CT identification of postlaminectomy pseudomeningocele. AJR Am. J. Roentgenol..

[11] Guerin P., El Fegoun A.B., Obeid I. (2012). Incidental durotomy during spine surgery: incidence, management and complicationsA retrospective review. Injury.

[12] Srilomsak P., Okuno K., Sakakibara T., Wang Z., Kasai Y. (2012 July). Giant pseudomeningocele after spinal surgery: a case report. World J. Orthop..

[13] Hussein M., Abdellatif M. (2019). Continuous lumbar drainage for the prevention and management of perioperative cerebrospinal fluid leakage. Asian J. Neurosurg..

[14] Maycock N.F., van Essen J., Pfitzner J. (1994). Post-laminectomy cerebrospinal fluid fistula treated with epidural blood patch. Spine.

[15] Berroir S., Loisel B., Ducros A. (2004 Nov). Early epidural blood patch in spontaneous intracranial hypotension. Neurology.

[16] Weng Y.J., Cheng C.C., Li Y.Y., Huang T.J., Hsu R.W. (2010). Management of giant pseudomeningoceles after spinal surgery. BMC Musculoskelet. Disord..

[17] Hawk M.W., Kim K.D. (2000 Jul). Review of spinal pseudomeningoceles and cerebrospinal fluid fistulas. Neurosurg. Focus..

[18] Tafazal S.I., Sell P.J. (2005). Incidental durotomy in lumbar spine surgery: incidence and management. Eur. Spine J..

[19] Muszbek L., Bereczky Z., Bagoly Z., Komáromi I., Katona É. (2011 Jul). Factor XIII: a coagulation factor with multiple plasmatic and cellular functions. Physiol. Rev..

